# 

**Published:** 1833-01

**Authors:** 


### LONDON MEDICAL AND PHYSICAL JOURNAL.

EDITED BY

JOHN NORTH, Esq. F.L.S.

MEMBER OF THE ROYAL COLLEGE OF SURGEONS, AND OF THE MEDICAL AND CHIRURGICAL SOCIETY OF LONDON;

AND

GILBERT T. BURNETT, Esq.

F.L.S. M.B. R.I. R.C.S. &c.

PROFESSOR OF BOTANY IN KING'S COLLEGE, LONDON, AND TO THE MEDICO-BOTANICAL SOCIETY.

(VOL. LXIX.)

NEW SERIES, VOL. XIV.

Imp.

Nos 145–152 gone.

Et quoniam variant morbi, variabimus artes;

Mille mali species, mille salutis erunt.

45602

S.G.O. INDEXED

LONDON:

JOHN SOUTER, 73, ST. PAUL'S CHURCH-YARD;

AND TO BE HAD OF ALL MEDICAL BOOKSELLERS.

1833.

W1
L031

J. and C. Adlard, Printers,
Bartholomew Close.

### MONTHLY LIST OF MEDICAL BOOKS.

[*Medical Works cannot be entered on this List unless a copy be sent for the purpose, the titles of Books having frequently been sent to us as published which have not appeared for weeks, or even months, after.*]

The Elements of Anatomy, by Jones Quain, b.m., Professor of Anatomy and Physiology in the University of London. Second Edition, *revised and corrected.*—8vo. pp. 812. John Taylor, London.

We announced the receipt of this excellent work in our last number, and we again place it on our list, for the purpose of informing those who have purchased it that, in consequence of several errors having accidentally passed uncorrected, some sheets have been cancelled. The publisher will be glad to exchange, without any additional charge, the uncorrected for the corrected copies; the latter may be known by the words "*revised and corrected,*" which have been inserted in the title page.

The Cyclopædia of Practical Medicine. Edited by John Forbes, m.d., f.r.s., &c., Alex. Tweedie, m.d., &c., and John Conolly, m.d., &c. Part XII. (published in Monthly Parts,) December 1832. Containing *Indigestion* (continued), by Dr. Todd; *Induration*, Dr. Carswell; *Infanticide*, Dr. Arrowsmith; *Infection*, Dr. Brown; *Inflammation*, Dr. Adair Craufurd, Dr. Tweedie.—Royal 8vo. pp. 107. Sherwood, Gilbert, and Piper; and Baldwin and Cradock, London.

A Treatise on the Urethra; its Diseases, especially Stricture, and their Cure. By Benj. Phillips.—8vo. pp. 317. Longman, London.

Elements of Surgery. By Robert Liston, Esq. Part III.—8vo. pp. 409. Longman, London; Black, Edinburgh.

A System of Materia Medica and Pharmacy, including Translations of the Edinburgh, London, and Dublin Pharmacopœias. By John Murray, m.d. &c. Sixth Edition, adapted to the present State of Chemical and Medical Science, by John Murray, m.d., f.r.c.s. e., &c.—8vo. pp. 886. Black, Edinburgh; Longman, London.

A much improved edition of a work which, even in its former state, had deservedly obtained an extensive circulation and great celebrity.

January 1st, No. I, The Principles and Illustrations of Morbid Anatomy, adapted to the Elements of M. Andral, and to the Cyclopædia of Practical Medicine, (with which it will correspond in size.) Being a complete Series of coloured Lithographic Drawings, from originals by the Author; with Descriptions, and summary Allusions to Cases, Symptoms, Treatment, &c. Designed to constitute an Appendix to Works on the Practice of Physic, and to facilitate the Study of Morbid Anatomy in connexion with Symptoms. By J. Hope, m.d., f.r.s., &c.—Large 8vo. pp. 23. Whittaker and Co., London.

A Short Statement, showing that, if the Allotment System were adopted, there would be no Excess of Population, no Necessity for Emigration, nor cultivating Waste Lands, nor for the Importation of Food. By J. Poole, Comm. r.n.—8vo. pp. 27. Westley, London.

A Treatise on the Diseases of the Liver and on Bilious Complaints; with Observations on the Management of the Health of those who have returned from Tropical Climates, and on the Diseases of Infancy. By G. H. Bell, f.r.c.s. e., &c.—8vo. pp. 152. Longman.

**The Physician's Vade Mecum; or, a Manual of the Principles and Practice of Physic: containing the Symptoms, Causes, Diagnosis, Prognosis, and Treatment of Diseases; accompanied by a select Collection of Formulæ, with a Table of the Doses of all Medicines now in use. By Robert Hooper, M.D. &c. New Edition, considerably enlarged and improved. 12mo. pp. 400. Renshaw and Rush, London.**

**An excellent book of reference for students and junior practitioners.**

---

### APOTHECARIES' HALL.

**Names of Gentlemen to whom the Court of Examiners have granted Certificates:**

**NOV. 29.**

Richard Hargrave Brett
John Doughty Brookes, Wenlock, Salop
William Blathwayt, Louth
Frederick Chambers, Hackney road
Robert Dade, Great Yarmouth
Clement Edkins, Dorking, Surrey
John Foote, Tavistock street
Robert Hawdon, Morpeth

**DEC. 6.**

Henry Bowen, St. Albans
Henry Stanhope Illingworth
William Salter, Whitchurch, Herefordshire
Robert Stopford Taylor, Sheffield

**DEC. 13.**

Alexandor Angus, Greek street, Soho
John Crea, Whittingham
Robert Davis, George street, New road
Henry Goddard
Alfred Higgenson, Derby
James Henly Hicks, Plymouth

**DEC. 20.**

Charles John Brickwell, Banbury
George Alfred Davenport, Market Harboro'
John Enscoe
John Hunt, Thumby, Leicestershire
Charles Potter, Brockhurst, Hants

**DEC. 28.**

John Carter, Coventry
William Hall, Leatherhead.

---

